# To infer the probability of cervical ossification of the posterior longitudinal ligament and explore its impact on cervical surgery

**DOI:** 10.1038/s41598-023-36992-7

**Published:** 2023-06-17

**Authors:** Jichong Zhu, Qing Lu, Xinli Zhan, Shengsheng Huang, Chenxing Zhou, Shaofeng Wu, Tianyou Chen, Yuanlin Yao, Shian Liao, Chaojie Yu, Binguang Fan, Zhenwei Yang, Wenfei Gu, Yihan Wang, Wendi Wei, Chong Liu

**Affiliations:** The First Affiliated Hospital of Guangxi Medical University, Nanning, 530021 People’s Republic of China

**Keywords:** Bone, Muscle

## Abstract

The ossification of the posterior longitudinal ligament (OPLL) in the cervical spine is commonly observed in degenerative changes of the cervical spine. Early detection of cervical OPLL and prevention of postoperative complications are of utmost importance. We gathered data from 775 patients who underwent cervical spine surgery at the First Affiliated Hospital of Guangxi Medical University, collecting a total of 84 variables. Among these patients, 144 had cervical OPLL, while 631 did not. They were randomly divided into a training cohort and a validation cohort. Multiple machine learning (ML) methods were employed to screen the variables and ultimately develop a diagnostic model. Subsequently, we compared the postoperative outcomes of patients with positive and negative cervical OPLL. Initially, we compared the advantages and disadvantages of various ML methods. Seven variables, namely Age, Gender, OPLL, AST, UA, BMI, and CHD, exhibited significant differences and were used to construct a diagnostic nomogram model. The area under the curve (AUC) values of this model in the training and validation groups were 0.76 and 0.728, respectively. Our findings revealed that 69.2% of patients who underwent cervical OPLL surgery eventually required elective anterior surgery, in contrast to 86.8% of patients who did not have cervical OPLL. Patients with cervical OPLL had significantly longer operation times and higher postoperative drainage volumes compared to those without cervical OPLL. Interestingly, preoperative cervical OPLL patients demonstrated significant increases in mean UA, age, and BMI. Furthermore, 27.1% of patients with cervical anterior longitudinal ligament ossification (OALL) also exhibited cervical OPLL, whereas this occurrence was only observed in 6.9% of patients without cervical OALL. We developed a diagnostic model for cervical OPLL using the ML method. Our findings indicate that patients with cervical OPLL are more likely to undergo posterior cervical surgery, and they exhibit elevated UA levels, higher BMI, and increased age. The prevalence of cervical anterior longitudinal ligament ossification was also significantly higher among patients with cervical OPLL.

## Introduction

The posterior longitudinal ligament (PLL) is a narrow ligament that extends along the posterior aspect of the vertebral bodies within the spinal canal. Arising from the body of the axis, PLL travels along the lamina covering the axis and gets attached to the sacrum. The PLL is more closely adherent with the annulus fibrosus of the intervertebral disc than at the vertebral body, where it is loosely connected, thus, limiting the excessive prodromal spinal movements. The simultaneous occurrence of ossification of the anterior longitudinal ligament (OALL), ossification of the posterior longitudinal ligament (OPLL), and ossification of the ligamentum flavum (OLF) is commonly observed in degenerative spine conditions. However, the exact frequency of this ossification remains unclear^1^. OPLL is a common degenerative disease of the entire spine; however, OPLL is more common in the cervical spine. The ossification and enlargement of ligaments can lead to compression of either the spinal cord or nerves, resulting in various neurological symptoms^2^. OPLL is most common in East Asians, with a reported prevalence of 1.9–4.3% in Japan and 0.1%–1.7% in Europe and the United States^3,4^. OPLL is more common in people aged 40–60 years, and the male-to-female ratio is 2:1^5^.

Cervical spine surgery is a common treatment for spinal cord nerve compression. There are several risks involved with cervical spine surgery; however, common complications include injuries to the nerves, airways, esophagus, large blood vessels, cerebrospinal fluid (CSF) leakage, wounds, and internal fixation fractures. Severe complications can also be life-threatening^6^. OPLL is an important factor that increases the risk of cervical spine surgery. Several studies have shown that the OPLL degree is significant in determining the effectiveness of clinicians’ surgical methods and techniques^7–9^. Therefore, it is very important to determine the cervical OPLL degree for choosing precise preoperative surgical methods and preventing postoperative complications.

Machine learning (ML) is a subdomain of artificial intelligence (AI) that provides the ability to fit predictive models to data or identify information groups in data centers. Moreover, it helps to approximate human pattern recognition in a more objective way^10^. Furthermore, ML algorithms can help humans identify and process the datasets without any manual intervention and can be further divided into two approaches: supervised learning and unsupervised learning. Processed datasets can either help in developing reliable risk models or re-classifying patients periodically^11^. Presently, ML methods are widely used in the medical field as they assist clinicians in diagnosing and treating diseases accurately^12^. We have also used ML methods for processing data related to ankylosing spondylitis and achieved good diagnostic efficiency^13^.

Few studies have used ML square regression method to construct a postoperative prediction model for OPLL patients for predicting postoperative improvement^14^. In this study, we collected the pre-and postoperative data of 752 patients undergoing cervical spine surgery for using the ML technique to predict the incidence of cervical OPLL and explore the postoperative differences between OPLL and other cervical spine surgery patients. This study aims to enhance our comprehension of the characteristics and distinctions of cervical OPLL.

## Patients and data collection

The patients who underwent cervical spine surgery from 2012 to 2021 at the First Affiliated Hospital of Guangxi Medical University were selected. Consent forms were signed by all patients, and for minor patients, the forms were signed by their guardians. (i) The study was approved by the Ethics Association of the First Affiliated Hospital of Guangxi Medical University. (ii) All research activities adhered to applicable guidelines and regulations. Patient privacy was safeguarded throughout the data processing and prior to the creation of raw files. We collected patients’ medical data, including erythrocyte sedimentation rate (ESR), high-sensitivity C-reactive protein (hs-CRP), blood routine, liver and kidney function tests, neurological function grading, medical history, imaging data, selection of surgical methods, operative time, intraoperative blood loss, postoperative drainage volume, and complications (Tables 1, 2, 3). The ligament ossification was judged by two clinicians by preoperative X-ray and computed tomography (CT) scan. The inclusion criteria for this study were as follows: (1) patients who underwent cervical spine surgery at the First Affiliated Hospital of Guangxi Medical University between 2012 and 2021; (2) patients with complete preoperative and postoperative imaging data available; (3) adult patients who provided voluntary consent and signed the informed consent form, while patients under 18 years old had their guardian sign on their behalf; (4) cervical spine surgery was determined to be the optimal treatment choice for the patient. The exclusion criteria encompassed: (1) patients with missing data; (2) patients unable to comply with pre- and post-treatment requirements as instructed by doctors and nurses; (3) patients with concurrent neurological disorders such as Parkinson’s disease or Alzheimer’s disease; (4) patients with severe cardiovascular, liver, or kidney diseases; (5) patients diagnosed with pneumonia, tuberculosis, emphysema, or other severe lung conditions; (6) patients affected by other serious illnesses, such as tumors.

**Table 1:** Differences in blood samples between cervical OPLL positive and negative patients

Type	Training cohort			Validation cohort		
	OPLL positive (N=107)	OPLL negative (N=509)	P-value	OPLL positive (N=37)	OPLL negative (N=122)	P-value
ESR						
Mean	21.75	18.6	0.107	18.35	17.52	0.793
WBC						
Mean	12.4	12.48	0.865	12.14	12.04	0.907
RBC						
Mean	4.29	4.33	0.601	4.37	4.37	0.999
HCT						
Mean	0.37	0.38	0.508	0.38	0.38	0.767
MCV						
Mean	87.56	87.61	0.956	88.18	87.73	0.786
MEHC						
Mean	28.9	28.89	0.984	29.04	28.997	0.95
MCHC						
Mean	329.54	329.23	0.805	328.8	329.8	0.668
PLT						
Mean	245.71	240.94	0.526	238.16	230.33	0.507
PDW						
Mean	0.155	0.154	0.729	0.156	0.156	0.968
NEUT#						
Mean	10.61	10.62	0.988	10.38	10.06	0.719
NEUT						
Mean	0.83	0.823	0.64	0.823	0.804	0.496
LYM#						
Mean	1.2	1.26	0.41	1.21	1.37	0.269
LYM						
Mean	0.114	0.122	0.455	1.223	0.136	0.51
MONO#						
Mean	0.495	0.505	0.798	0.477	0.511	0.643
MONO						
Mean	0.043	0.44	0.821	0.043	0.047	0.539
EO#						
Mean	0.08	0.078	0.937	0.064	0.084	0.495
EO						
Mean	0.01	0.009	0.452	0.009	0.01	0.752
BASO						
Mean	0.02	0.021	0.846	0.017	0.024	0.221
CV						
Mean	0.136	0.137	0.824	0.138	0.135	0.246
PCT						
Mean	0.208	0.206	0.686	0.203	0.197	0.525
TBil						
Mean	11.85	11.43	0.446	11.27	11.4	0.897
DBil						
Mean	3.46	3.34	0.525	3.08	3.47	0.26
IBil						
Mean	8.4	8.12	0.505	8.19	8	0.804
DBil/TBil						
Mean	0.3	0.3	0.885	0.28	0.3	0.146
TB						
Mean	64.79	64.17	0.41	64.49	64.61	0.928
ALB						
Mean	37.49	37.65	0.704	37.51	38.29	0.29
GLB						
Mean	27.29	26.52	0.183	26.98	26.34	0.506
ALB/GLB						
Mean	1.42	1.48	0.083	1.45	1.49	0.444
GGT						
Mean	36.77	32.34	0.179	28.73	32.82	0.606
TBA						
Mean	3.8	4.43	0.561	5.27	3.1	**0.018**
AST						
Mean	29.73	24.33	**<0.001**	25.14	23.34	0.582
ALT						
Mean	28.8	22.49	**0.001**	28.57	26.14	0.761
AST/ALT						
Mean	1.28	1.44	0.395	1.05	1.21	0.242
ALP						
Mean	75.68	71.59	0.131	69.05	72.31	0.539
PAB						
Mean	236.78	240.68	0.536	243.32	242.24	0.91
ChE						
Mean	7912.2	7844.77	0.705	8251.4	7989.4	0.467
BUN						
Mean	5.42	5.12	0.113	4.92	4.9	0.949
Cr						
Mean	66.72	66.84	0.947	69.24	67.78	0.615
UA						
Mean	280.31	255.59	**0.012**	285.27	260.86	0.138
HCO						
Mean	23.46	24.17	**0.035**	23.62	24.46	0.115
Ccr						
Mean	106.42	105.23	0.697	101.42	107.96	0.263
Cys-C						
Mean	0.79	0.79	0.856	0.82	0.77	0.339

ESR, erythrocyte sedimentation rate,mm/h; WBC, White blood cell count, 109/L; RBC, Red blood cell count,1012/L; HCT, hematocrit value; MCV, Mean corpuscular volume, fl; MEHC, Mean erythrocyte hemoglobin content,pg; MCHC, Mean corpuscular hemoglobin concentration,g/l; PLT, Platelet hematocrit, 109/L; PDW, Platelet distribution width; NEUT, neutrophil percentage; LYM, lymphocytes percentage; MONO, Monocyte percentage; EO, eosinophils percentage; BASO, Absolute value of basophils; CV, RBC volume distributing width; PCT, thrombocytocrit.TBil, total bilirubin, μmol/L; DBil, direct bilirubin, μmol/L; IBil, indirect bilirubin, μmol/L; TP, total protein, g/L; ALB, albumin, g/L; GLB, globulin, g/L; GGT, gamma-glutamyl transpeptidase, U/L; TBA, Total bile acid, μmol/L; AST, aspartate aminotransferase, U/L; ALT, alanine aminotransferase, U/L; ALP, A alkaline phosphatase, U/L; PAB, prealbumin, mg/L; ChE, cholinesterase, U/L; BUN, Blood urea nitrogen, μmol/L; Cr, Creatinine, μmol/L; UA, Uric acid, μmol/L; HCO, Hicarbonate radical, mmol/L; Ccr, Creatinine clearance rate, ml/min; Cys-C, Cysteine C, mg/L.

Significant values are in bold.

**Table2:** Difference of clinical data before operation between cervical OPLL positive and negative patients

Type	Training cohort			Validation cohort		
	OPLL positive (N=107)	OPLL negative (N=509)	P-value	OPLL positive (N=37)	OPLL negative (N=122)	P-value
OALL						
Positive	29(27.1%)	35(6.9%)	**<0.001**	14(37.8%)	16(13.1%)	**0.001**
Negative	78(72.9%)	474(93.1%)		23(62.2%)	106(86.9%)	
Gender						
Male	38(35.5%)	291(57.2%)	**<0.001**	22(59.5%)	78(64%)	0.379
Female	69(64.5%)	218(42.8%)		15(40.5%)	44(36%)	
hs-CRP						
<0.8	31(29%)	182(35.8%)	0.317	18(48.6%)	47(38.5%)	0.539
0.8-10	48(44.9%)	220(43.2%)		13(35.1%)	53(43.4%)	
>10	28(26.1%)	107(21%)		6(16.2)	22(18.1%)	
Age						
Mean	56.8	54.48	**0.035**	56.92	55.38	0.438
BMI(kg/m2)						
Mean	24.2	23.24	**0.004**	23.5	23.45	0.919
Emergency admission						
Yes	2(1.9%)	5(1%)	0.35	1(2.7%)	2(1.6%)	0.551
No	105(98.1%)	504(99%)		36(96.3%)	120(98.4%)	
Classification of neural function						
3	46(43%)	199(39.1%)	0.26	15(40.5%)	45(36.9%)	0.414
4	61(57%)	310(60.9%)		22(59.5%)	77(63.1%)	
History of cervical spine surgery						
Yes	10(9.3%)	23(4.5%)	**0.044**	6(16.2%)	5(4.1%)	**0.02**
No	97(90.7%)	486(95.5%)		31(83.8%)	117(95.9%)	
Cervical instability						
Yes	18(16.8%)	75(14.7%)	0.338	4(10.8%)	12(9.8%)	0.537
No	89(83.2%)	434(85.3%)		33(89.2%)	110(90.2%)	
Diabetes						
Yes	9(8.4%)	24(4.7%)	0.1	2(5.4%)	9(7.4%)	0.506
No	98(91.6%)	485(95.3%)		35(94.6%)	113(92.6%)	
Hypertension						
Yes	29(27.1%)	78(15.3%)	**0.004**	3(8.1%)	26(21.3%)	0.051
No	78(72.9%)	431(84.7%)		34(91.9%)	96(78.7%)	
CHD						
Yes	3(3%)	2(0.4%)	**0.039**	1(3%)	1(0.8%)	0.412
No	104(97%)	507(99.6%)		36(97%)	121(99.2%)	
Abnormal liver and kidney function						
Yes	3(3%)	3(0.6%)	0.412	0(0%)	2(1.6%)	0.588
No	104(97%)	506(99.4%)		37(100%)	120(98.4%)	
Cerebrovascular Disease						
Yes	3(3%)	13(2.6%)	0.546	1(3%)	1(0.8%)	0.412
No	104(97%)	496(97.4)		36(97%)	121(99.2%)	
Peptic ulcer						
Yes	5(4.7%)	27(5.3%)	0.508	0(0%)	1(0.8%)	0.767
No	102(95.3%)	482(94.7%)		37(100%)	121(99.2%)	
Cancer						
Yes	0(0%)	5(1)	0.384	0(0%)	0(0%)	-
No	107(100%)	504(99%)		37(100%)	122(100%)	
Osteoporosis						
Yes	7(6.5%)	21(4.1%)	0.197	5(13.5%)	10(8.2%)	0.25
No	100(93.5%)	488(95.9%)		32(86.5%)	112(91.8%)	
AS						
Yes	1(1%)	2(0.4%)	0.436	0(0%)	1(0.8%)	0.767
No	106(99%)	507(99.6%)		37(100%)	121(99.2%)	
Rheumatoid arthritis						
Yes	3(3%)	7(1.4%)	0.244	0(0%)	1(0.8%)	0.767
No	104(97%)	502(98.6%)		37(100%)	121(99.2%)	
Preoperative VAS score						
Mean	3.12	3.51	0.131	4.03	3.3	**0.081**
Cigarette						
Yes	17(15.9%)	79(15.5%)	0.5512	4(10.8%)	21(17.2%)	0.255
No	90(84.1%)	430(84.5%)		33(89.2%)	101(82.8%)	

OALL, Ossification of the anterior longitudinal ligament. hs-CRP, Hypersensitive C-reactive protein; BMI, Body Mass Index; CHD, coronary heart disease; AS, Ankylosing spondylitis;

Significant values are in bold.

**Table 3:** Differences in postoperative clinical data between cervical OPLL positive and negative patients

Type	Training cohort			Validation cohort		
	OPLL positive (N=107)	OPLL negative (N=509)	P-value	OPLL positive (N=37)	OPLL negative (N=122)	P-value
Surgical method						
Anterior operation	74(69.2%)	442(86.8%)	**<0.001**	27(73.0%)	103(84.5%)	0.14
Posterior operation	31(29.0%)	57(11.2%)		10(27.0%)	17(13.9%)	
Anterior and posterior operation	2(1.8%)	10(2%)		0(0%)	2(1.6%)	
Decompression segment of spinal canal						
1	7(6.5%)	47(9.2%)	**<0.001**	5(13.5%)	16(13.1%)	**0.046**
2	49(45.8%)	307(60.3%)		15(40.5%)	68(55.7%)	
3	30(28%)	122(24%)		7(18.9%)	27(22.1%)	
4	17(15.9%)	27(5.3%)		10(27%)	10(8.2%)	
5	4(3.7%)	6(1.2%)		0(0%)	1(0.8%)	
Time of operation(min)						
Mean	106.8	93.46	**<0.001**	103.19	97.99	0.451
Intraoperatve blood soss						
Mean	193.1	180.5	0.582	309.19	247.87	0.548
Blood transfusion volume						
Mean	16.8	15.1	0.853	140.54	118.85	0.795
Postoperative drainage						
Mean	191.7	119.1	**<0.001**	168.97	138.53	0.36
Hospital stay						
Mean	8.56	8.26	0.442	8.59	8.66	0.928
Postoperative improvement rate of JOA						
Mean	55.19	65.6	**0.003**	65.36	69.9	0.303
Postoperative dyspnea						
Yes	1(0.9%)	2(0.4%)	0.436	0(0%)	0(0%)	-
No	106(99.1%)	507(99.6%)		37(100%)	122(100%)	
Postoperative digestive tract ulcer						
Yes	1(0.9%)	2(0.4%)	0.436	0(0%)	2(1.6%)	0.58
No	106(99.1%)	507(99.6%)		37(100%)	120(98.4%)	
Postoperative dysphagia						
Yes	1(0.9%)	4(0.8%)	0.616	0(0%)	1(0.8%)	0.762
No	106(99.1%)	505(99.2%)		37(100%)	121(99.2%)	
Pneumonia						
Yes	4(3.7%)	11(2.2%)	0.254	0(0%)	1(0.8%)	0.762
No	103(96.3%)	498(97.8%)		37(100%)	121(99.2%)	
Hoarseness after surgery						
Yes	0(0%)	4(0.8%)	0.254	0(0%)	0(0%)	-
No	107(100%)	505(99.2%)		37(100%)	122(100%)	
Postoperative psychosis						
Yes	1(0.9%)	3(5.9%)	0.535	0(0%)	0(0%)	-
No	106(99.1%)	506(94.1%)		37(100%)	122(100%)	
Postoperative nerve paralysis						
Yes	4(4%)	5(1%)	0.054	3(8.1%)	6(4.9%)	0.363
No	103(96%)	504(99%)		34(91.9%)	116(95.1%)	
Axial pain after surgery						
Yes	1(0.9%)	5(1%)	0.72	0(0%)	0(0%)	-
No	106(99.1%)	504(99%)		37(100%)	122(100%)	
leakage of cerebrospinal						
Yes	7(6.5%)	10(2%)	**0.017**	0(0%)	2(1.6%)	0.58
No	100(93.5%)	499(98%)		37(100%)	120(98.4%)	
Esophageal fistula						
Yes	0(0%)	2(0.4%)	0.683	0(0%)	1(0.8%)	0.762
No	107(100%)	507(99.6%)		37(100%)	121(99.2%)	
Local hematoma formed						
Yes	0(0%)	4(0.8%)	0.465	0(0%)	0(0%)	-
No	107(100%)	505(99.2%)		37(100%)	122(100%)	
IRI						
Yes	6(5.6%)	6(1.2%)	0.009	0(0%)	0(0%)	-
No	101(94.4%)	503(98.8%)		37(100%)	122(100%)	
Incision infection						
Yes	2(1.8%)	3(0.6%)	0.209	0(0%)	1(0.8%)	0.762
No	105(98.2%)	506(99.4%)		37(100%)	121(99.2%)	
Use antibiotics beyond the perioperative period						
Yes	9(8.4%)	29(5.7%)	0.197	1(2.8%)	5(4.3%)	0.363
No	98(91.6%)	480(94.3%)		36(97.2%)	117(95.7%)	

IRI, schemia and reperfusion injury. JOA, Japanese Orthopaedic Association.

Significant values are in bold.

A cohort of 775 patients who underwent cervical spine surgery at the Department of Spine and Orthopedic Surgery, the First Affiliated Hospital of Guangxi Medical University, was included in this study. Among them, 144 patients had cervical spine with OPLL, while 631 patients did not. The training cohort consisted of 107 patients with positive cervical OPLL and 509 patients with negative cervical OPLL. In the validation cohort, there were 37 patients with OPLL positive and 122 patients with OPLL negative.

### Statistical analysis

We utilized IBM SPSS 23.0 software (IBM, Armonk, NY, USA) for data analysis. Before the continuous variable data analysis, we tested the normal distribution of data and the homogeneity of variance test. The data with normal distribution and homogeneity of variance were tested by t-test or the Mann-Whitney U test. We used the chi-square test for comparing two data groups for constituent ratios or two different rates, and Fisher’s exact test was used in small samples. After completing the analysis, *p*<0.05 denoted statistical significance.

Visual image generation and analysis were performed using the R software (R 4.2.2; https://www.R-project.org). Lasso regression analysis was performed by using the “Glmnet” package of R software. In Fig. 2, The R package functions like “caret”, “DALEX”, “ggplot2”, "randomForest", "kernlab", "xgboost", and "pROC" were used for aiding Random Forest (RF) and Generalized Linear models based on four machine methods. Other models like (GLM), XGboost (XGB), and support vector machine (SVM) were used for data analysis and visualization^15,16^. We used the R packages "rms" and "regplot" to establish a nomogram^17^. Additionally, the R package "rms" was also applied to decision *curve* analysis (DCA) and the construction of fitted curves.

### Ethics statement

(i)The Ethics Association of the First Affiliated Hospital of Guangxi Medical University approved this study (Supplementary Material 3). (ii) All studies were conducted in accordance with relevant guidelines and regulations. In order to protect the privacy of patients, the ID and name of patients were concealed during data collection and collating.

## Results

Table 1 shows the differences in blood routine, liver, and kidney function tests of all the patients. Although there was no significant difference in most of the blood samples, the aspartate aminotransferase (AST) (29.73U/L), alanine aminotransferase (ALT) (28.8 U/L), and uric acid (UA) (280.31μmol/L) levels in cervical OPLL-positive patients in the training and validation groups were higher than the cervical OPLL-negative patients; the difference was statistically significant. The mean bicarbonate (HCO) (23.46mmol/L) in renal function of cervical OPLL-positive patients was lower than the patients with negative cervical OPLL, with a statistically significant difference as displayed in Table 1.

Table 2 presents the preoperative data of patients who underwent cervical spine surgery. The findings revealed a significantly higher prevalence of cervical OPLL among female patients (64.5%) compared to male patients (35.5%) undergoing surgery for cervical spondylosis. Furthermore, the average age and body mass index (BMI) of patients with positive cervical OPLL (24.2 kg/m^2^) were higher than those without cervical OPLL (23.24 kg/m^2^), with statistical significance observed in the training group. Additionally, patients with a history of previous cervical spine surgery demonstrated an increased risk of developing cervical OPLL (9.3%). Moreover, hypertensive patients had a higher risk of cervical OPLL (27.1%), although opposite results were found in the training and validation cohorts, respectively. We suggest that this might be due to a randomization error. The data suggested a higher incidence of OPLL in patients with coronary heart disease (CHD) (3%), but the sample size was small. In both training and validation cohorts, patients with positive cervical anterior longitudinal ligament (OALL) were more likely to develop OPLL (27.1%).

### Prediction of the OPLL model

**Figure 1:**
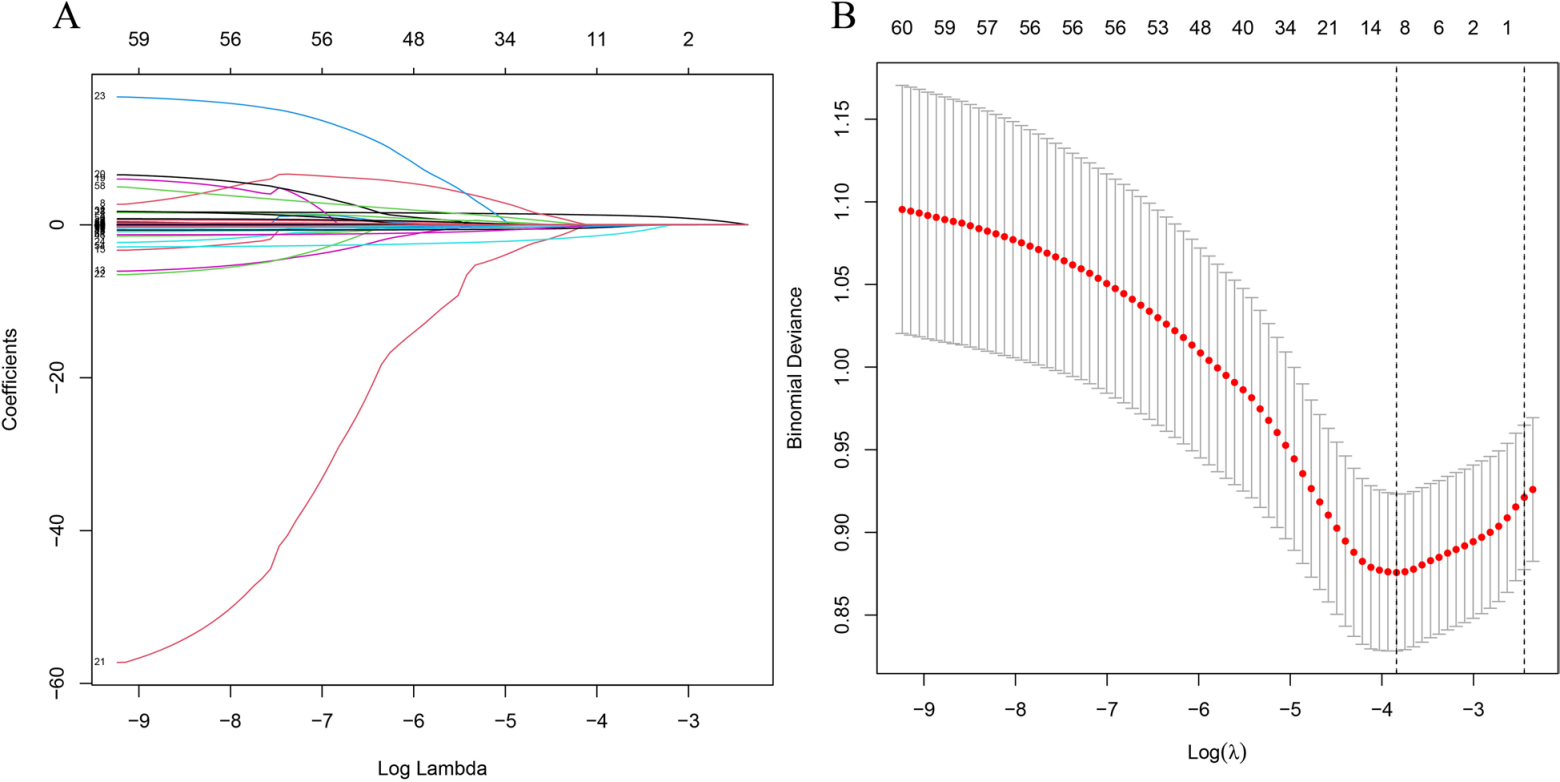
All preoperative factors of OPLL patients were analyzed by Lasso regression. **(A)** LASSO regression for dependent variables. **(B)** Ten variables were significantly different between OPLL positive and negative cases.

**Figure 2:**
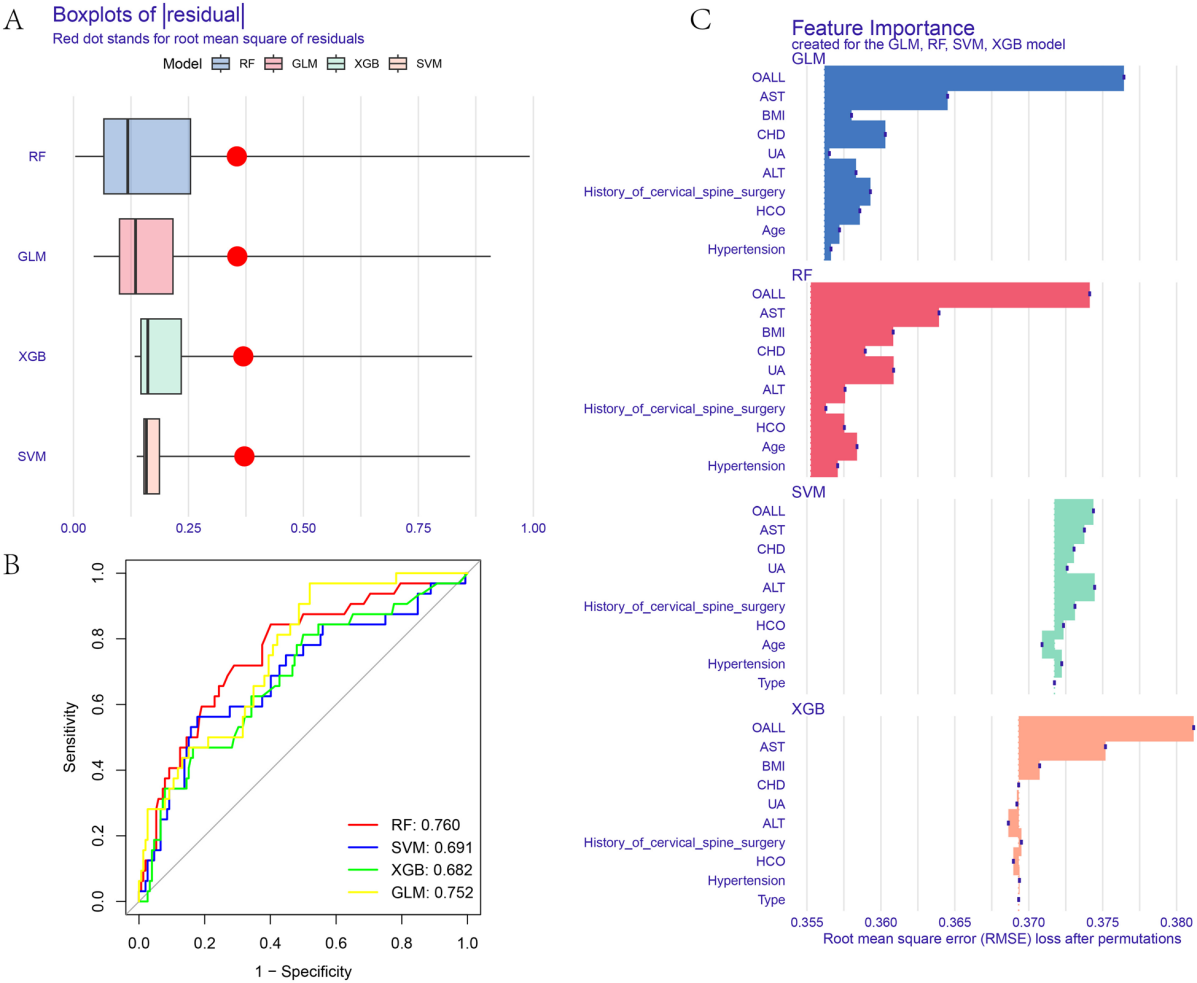
The RF, GLM, XGB, and SVM methods were used to screen the data again. **(A)** Residual values for RF, GLM, XGB, and SVM. **(B)** AUC values for the ML model. **(C)** Importance ranking of each factor in the RF, GLM, XGB, and SVM models.

Due to the extensive number of variables, we initially employed the Lasso regression method to screen the fundamental data in Table 1 of patients undergoing cervical spine surgery. Fig. 1A and B demonstrate that the Lasso regression yielded 10 factors: OALL, Age, AST, ALT, UA, HCO, BMI, History of cervical spine surgery, Hypertension, and CHD. These 10 factors were subsequently utilized for further analysis. Supplementary material 1 presents a correlation diagram illustrating the relationships among the 10 factors. Notably, no significant relationships were observed among these factors, indicating a relatively high level of independence among them. Four ML methods, RF, GLM, XGB, and SVM, were used for further data screening. Figure 2A shows a boxplot of the residuals for the four ML methods. The lower the residuals, the higher the accuracy of the model. Figure 2B shows the receiver operating characteristic curves (ROC) of the four ML, and the higher the area under the curve (AUC), the higher the diagnostic efficiency of the ML. Figure 2C shows the ranking of importance of each factor in different ML. The longer the bar chart, the higher the importance of the factor in this ML. In the prediction of cervical OPLL, logistic regression model was employed, resulting in the inclusion of four factors: CHD, AST, History of cervical spine surgery, and OALL (Supplementary Material 4). The diagnostic model based on these factors achieved an AUC value of 0.6830692 (Supplementary Material 5). Among the five ML methods considered, RF exhibited the best diagnostic performance.

**Figure 3:**
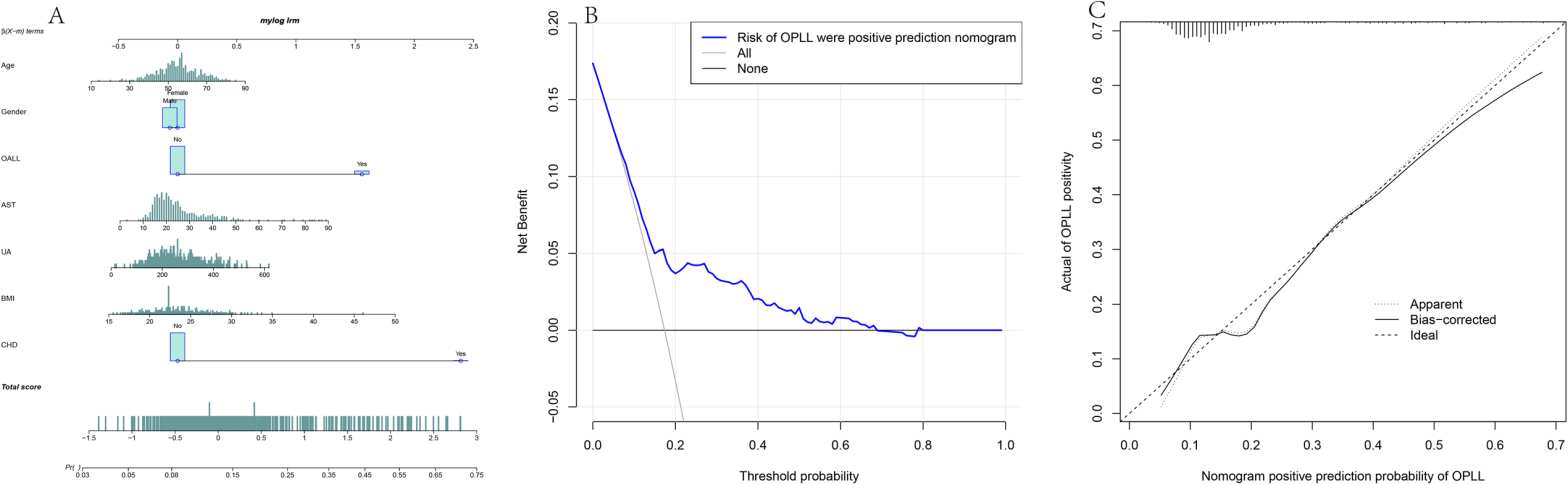
The top 7 factors screened by RF were used to construct the diagnostic model. **(A)** Nomogram diagnostic model. **(B)** Decision curve analysis showed the OPLL model. **(C)** Calibration curves for prediction of OPLL probability.

**Figure 4:**
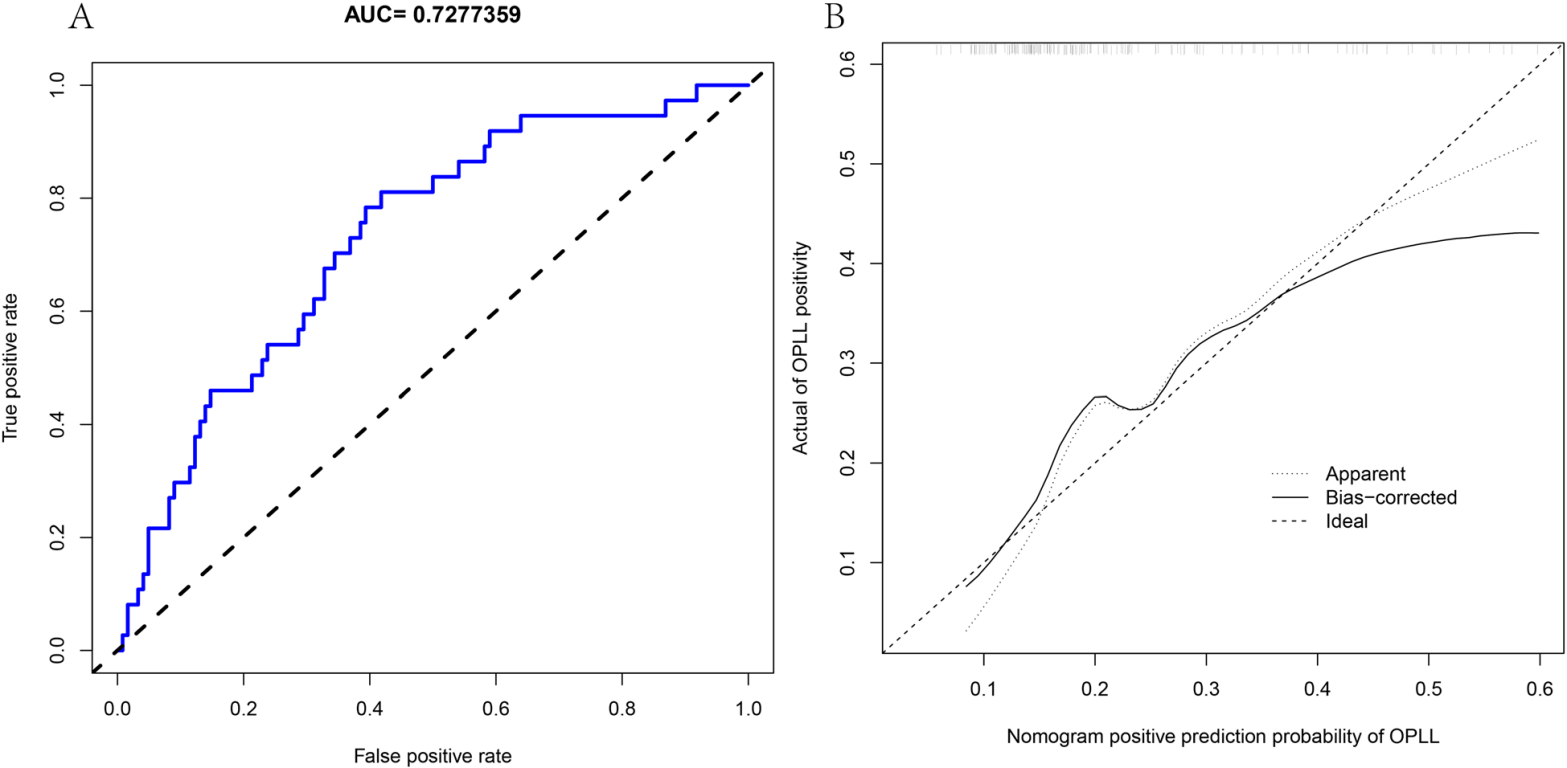
The diagnostic model was validated in the validation cohort. **(A)** AUC values of the diagnostic model in the validation cohort. **(B)** Calibration curves of the diagnostic model in the validation cohort.

Following multiple tests and calculations in the training and validation cohorts, considering the higher proportion of female patients with cervical OPLL, we ultimately included the top 6 factors from RF, as well as gender, in the construction of the diagnostic model. These factors were Age, Gender, OALL, AST, UA, BMI, and CHD. To determine the positive rate of OPLL, we developed a nomogram, as depicted in Fig. 3A. Figure 3B presents a decision curve analysis, examining the clinical utility range of our model. The decision curve consistently showed higher values for the ALL line compared to the NONE line, with a threshold range of 5% to 69%. Furthermore, the calibration curve (Fig. 3C) illustrates the relationship between the actual incidence and the predicted incidence, with calibration data plotted on the curve. The calibration curve demonstrates the accurate prediction of OPLL occurrence. Figure 4 is our validation in the validation queue. Figure 4A suggests that the AUC value of our diagnostic model in the validation queue is 0.7277359. Figure 4B shows that the diagnostic accuracy of the diagnostic model is still good in the validation queue.

### Postoperative differences in patients with cervical OPLL

**Figure 5:**
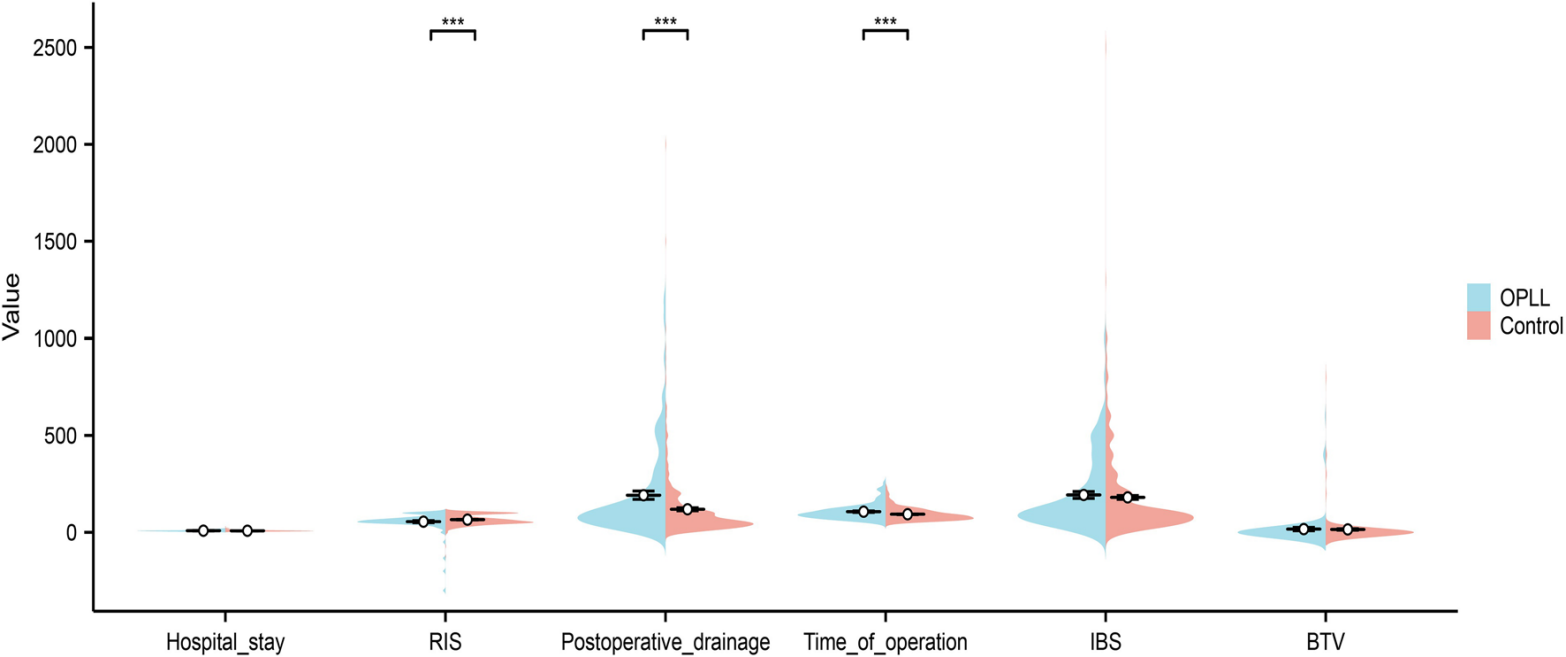
The postoperative data of OPLL positive and negative patients were compared.

Following the development of a nomogram for predicting OPLL, our objective was to investigate the disparities between patients with positive and negative OPLL following cervical spine surgery. To achieve this, we collected a substantial amount of postoperative data related to the cervical spine, as illustrated in Table 3. The findings from Table 3 indicate that patients with positive cervical OPLL were more inclined to undergo posterior cervical surgery (29.0%) and multi-level cervical surgery (93.5%). Additionally, there was a significant increase in the incidence of cerebrospinal leakage (6.5%) and ischemia reperfusion injury (IRI) (5.6%). To further explore the differences in postoperative continuous variables between OPLL-positive and OPLL-negative patients, we constructed a pod map. Both Table 3 and Fig. 5 highlight that patients with positive cervical OPLL exhibited prolonged operation time (106.8 minutes), increased postoperative drainage volume (191.7 ml), and a significantly lower improvement rate according to the Japanese Orthopaedic Association (JOA) operative score (55.19).

## Discussion

OPLL is a multifactorial disease with an idiopathic nature, and it is known to have a certain degree of familial heritability. The pathogenesis of OPLL involves factors such as diet, obesity, posterior longitudinal ligament injury or fatigue, age, and diabetes^18^. Detecting OPLL in its early stages poses challenges, and some patients with confirmed posterior longitudinal ligament injury may not exhibit significant changes in clinical symptoms. Surgeons rely on physical examinations or neurologic-related assessments to identify signs of changes, such as the Hoffman reflex, gait alterations, and muscle strength variations^8^. Currently, the diagnosis of OPLL primarily depends on CT scans or lateral X-ray films, which offer higher accuracy^19^. When OPLL patients present symptoms of nerve compression, MRI examinations are necessary to determine the location of the compression^20^. In this study, we analyzed the data of patients with cervical OPLL to find the relevant differences between patients with positive and negative cervical OPLL and constructed a diagnostic model using the ML method. To a certain extent, it can help clinicians diagnose cervical OPLL and find out the differences. It also provides a new research direction for the prevention and treatment of OPLL in the future.

Table 1 displays the variations in blood samples between patients with positive and negative cervical OPLL. Although the mean erythrocyte sedimentation rate (ESR) was higher in cervical OPLL-positive patients in both the training and validation cohorts, the difference did not reach statistical significance. Previous studies have indicated a positive correlation between serum insulin, osteocalcin, and OPLL^21,22^. In recent studies involving patients treated for breast cancer and rheumatoid arthritis, a reverse relationship has been observed between serum insulin, osteocalcin, and ESR^23,24^. However, in our study, we did not measure insulin and osteocalcin levels in patients, leaving the cause for the elevated ESR in cervical OPLL-positive patients unknown. In the liver function test, the mean AST and ALT of cervical OPLL-positive patients were higher than the negative patients. Both AST and ALT are widely present in the human body, especially in liver mitochondria. However, in cases of increased liver function load or abnormal liver function, AST and ALT increase partly. Moreover, few studies have linked OPLL with liver function metabolism, which is a discovery for us. It has been shown that sclerostin, a glycoprotein, significantly affects bone formation and is relatively elevated in OPLL-positive patients^25^. Another study reported that sclerostin is positively correlated with liver function and BMI in patients with excessive alcohol consumption^26^. Our results showed that BMI was higher in cervical OPLL-positive patients than in cervical OPLL-negative patients. Therefore, we suggest that sclerostin may increase AST and ALT levels in OPLL patients, but further studies are needed to validate it. In another study, OPLL-induced mechanical emergency of ligament tissue increased alkaline phosphatase (ALP) expression^27^. A similar result was obtained in our study, but there was no statistical difference in ALP elevation.

A statistical difference was observed between UA and HCO in renal function tests; UA was significantly elevated in cervical OPLL-positive patients. UA is a heterocyclic compound containing carbon, nitrogen, and other substances, which is eliminated by glomerular filtration. However, the imbalance of UA production and elimination causes elevated uric acid in the blood, thus, causing gout. Common causes of increased UA are over intake of purines, overproduction of endogenous purines, or increased purine metabolism. It has been shown that UA increases significantly during the progression of OPLL^28^. Although diffuse idiopathic osteosis is also associated with post-osseous osteoporosis and hyperuricemia^29^, the direct relationship between OPLL and hyperuricemia has not been clearly reported.

Among 775 patients undergoing cervical spine surgery, 144 were patients with OPLL, accounting for 18.6% of the total. Age plays an important role in cervical OPLL. The average age of onset of OPLL was 51 and 49 years old for males and females, while 67% of the patients were 45–65 years old, respectively^30^. With advancing age, the range of OPLL hyperplasia increases correspondingly. In our study, the average age of cervical OPLL-positive patients was higher than the negative patients. The mean age of patients undergoing cervical OPLL surgery was 56.8. Clinically, most patients' willingness to undergo cervical spine surgery is closely related to the presenting symptoms. It has been proved that the older the cervical spine deformity, the worst the postoperative recovery^31^. Our results showed that patients with positive cervical OPLL had poor postoperative recovery, as judged by the postoperative improvement score of JOA. Hence, early detection of cervical OPLL in the patient might lead to timely surgical intervention and account for better results.

Gender is an influential factor in patients with cervical OPLL. Our study revealed a higher proportion of cervical OPLL patients among women, with 84 female patients accounting for 58% of the total, while male patients constituted 42% of the OPLL cases. There are several potential reasons for this disparity. Firstly, women have inherent biological differences from men, including lower bone density and softer ligaments. These physiological distinctions may contribute to a higher likelihood of posterior longitudinal ligament ossification in female patients. Secondly, the decline in estrogen levels after menopause can lead to decreased bone density and weakened ligaments, thereby increasing the risk of posterior longitudinal ligament ossification. Our viewpoint is supported by other epidemiological studies on OPLL^32^. Considering the elevated incidence of OPLL in female patients and the ease of obtaining gender information as a variable, we also incorporated gender into our diagnostic model. However, it is important to note that the patients included in our study were part of a retrospective analysis following cervical spine surgery, which may introduce research biases and cannot be representative of an epidemiological investigation. BMI is an important international standard used to measure obesity. As previously mentioned, cervical OPLL-positive patients have a higher BMI than cervical OPLL-negative patients, thus, indicating the correlation of obesity with OPLL. A Japanese study confirmed that BMI is associated with the prevalence of OALL and OPLL, as obesity influences the development of ectopic spinal ligament ossification^31^. Suggesting a relationship between obesity and leptin secretion, another study demonstrated that abnormal leptin secretion could directly act on mesenchymal stem cells to down-regulate bone formation and promote bone marrow adipogenesis^33^.

The pathogenesis of cervical OALL is similar to that of OPLL. Clinically, OALL is more prevalent than OPLL. The incidence of OALL is about 6.6%, 19.15%, and 1.95% in the cervical spine, thoracic spine, and lumbar spine, respectively^34^. Both cervical OALL and OPLL affect cervical stability and function. Like our OPLL study, OALL has been associated with obesity, type 2 diabetes, and advancing age^35,36^. The higher incidence of OALL corresponds to its rapid progression. Since the incidence of cervical OALL is higher than that of OPLL, it is necessary to consider OPLL as a diagnosis when imaging data suggest OALL^1^.

CHD was also included in our diagnostic model, but the number of CHD cases was too low to be a very convincing reference. As bone metabolism depends on the balance between osteoblasts and osteoclasts, it has been suggested that bone mass decreases swiftly in patients with increased severity of aortic calcification^37^. The etiology of OPLL and the mechanism of vascular calcification in CHD have not been studied appropriately. Also, no relevant epidemiological investigations have assessed whether CHD patients have a higher incidence of OPLL. We suggest that further studies should validate this aspect in the near future.

The treatment of cervical OPLL can be divided into conservative and surgical interventions. The conservative treatment of OPLL mainly includes bed rest, brace fixation, pain relief, and blood supply improvement for myelopathy or nerve root symptoms. At present, there is no effective method to prevent OPLL progression, and it may even increase the risk of spinal cord injury^38^. In the presence of severe spinal cord lesions or spinal stenosis, cervical spine surgery can be considered for radical treatment. A few surgical methods include anterior decompression and fusion surgery, posterior decompression surgery, as well as a combined anterior and posterior decompression surgery. The specific surgical procedure is decided after communication between the doctor and the patient. In our study, the proportion of cervical OPLL patients undergoing posterior decompression surgery was significantly higher, with a higher probability of multi-level decompression. Since cervical OPLL involves more vertebral segments and has a tendency to progress, posterior decompression surgery can achieve sufficient decompression and is easy to operate^39^.

When cervical OPLL patients underwent surgery, their operating time and postoperative drainage volume were higher than the patients without OPLL. The operating time is closely related to the degree of difficulty of the operation, and the postoperative drainage volume is related to the severity of intraoperative trauma. In OPLL patients, the hyperplastic bone often increases the difficulty of decompression of the spinal cord, and its dissection can easily damage the spinal cord and cause CSF leakage^40^. In our study, OPLL-positive patients had a 4.9% chance of developing a CSF leakage, whereas patients with negative OPLL had a 1.9% chance of developing a CSF leakage. The incidence of IRI in cervical OPLL patients was also higher and may be related to severe compression of the spinal cord caused by OPLL. Hence, surgeons should consider the risk of IRI when facing spinal cord compression and degeneration caused by severe OPLL.

The application of ML in clinical data processing presents several advantages. ML algorithms enable rapid and accurate analysis of large volumes of clinical data, leading to precise diagnoses and treatment plans, as well as reducing the time required for data analysis and processing. Moreover, ML can analyze individual patient data and generate personalized treatment plans tailored to specific needs. It can also predict the occurrence and progression of diseases based on personalized data in a timely^12^. However, ML does have its limitations. The effectiveness of ML relies on the quality of the training data used. Incomplete or significantly flawed data can result in inaccurate outcomes. Additionally, while ML is highly efficient, interpreting and comprehending its results can be challenging, making it difficult for clinicians to fully trust the outcomes produced by ML^11^.

Since ML algorithms are increasingly being used in medical fields, several other studies have applied ML to validate the aspects of OPLL. For example, ML can predict C5 nerve palsy after cervical OPLL and to predict postoperative improvement of OPLL^14,41^. We collected 775 cervical spine surgery patients’ data and 84 complete variables to screen out relevant characteristics of cervical OPLL and construct a diagnostic model. For instance, in cases where a patient's cervical CT examination is lacking or small OPLL cannot be detected by X-ray, the utilization of a nomogram can aid in predicting cervical OPLL to some extent, thereby assisting doctors in optimizing surgical procedures. When performing cervical spine surgery on patients with positive OPLL, physicians should exercise caution during intraoperative procedures to prevent surgery-related complications. Furthermore, careful attention should be given to postoperative fluid replacement. It is advisable to employ a wound drainage bag after surgery and continuously monitor the postoperative drainage volume of patients. Additionally, our findings have uncovered potential correlations between OPLL and other factors, presenting promising avenues for future research. Our study had a few limitations. Firstly, our data were from a single center, and although they were grouped internally, they were less convincing. Secondly, the performance of our diagnostic model for cervical OPLL was not ideal, and lastly, we did not compare the diagnostic efficacy of all available ML techniques; only the efficacy of common centralized ML methods was evaluated.

## Conclusion

In summary, we constructed a diagnostic model of cervical OPLL using the ML method and analyzed the postoperative differences in OPLL-positive patients. Our findings provide new techniques and insights for streamlining the diagnosis, treatment, and research direction of OPLL in the future.

## Supplementary Information

Supplementary Information 1.

Supplementary Information 2.

Supplementary Information 3.

Supplementary Information 4.

Supplementary Information 5.

Supplementary Information 6.

## Data Availability

Data collected in the study are in the article/Supplementary Material 2; please contact the corresponding author for further inquiries.
