# Supplementary material for: To infer the probability of cervical ossification of the posterior longitudinal ligament and explore its impact on cervical surgery
Source: Sci Rep. 2023 Jun 17;13:9816. doi: 10.1038/s41598-023-36992-7 (PMC10276809; doi:10.1038/s41598-023-36992-7)
Supplement: Supplementary file 1 — Supplementary Information 1. [file 41598_2023_36992_MOESM1_ESM.docx]

**Supplementary Materials**

Supplementary Material 1 is the correlation diagram. Supplementary Material 2 is the original data of the article. Supplementary Material 3 is the Ethical review document.

Supplementary Material 4 is the logistic regression result. Supplementary Material 5 is the AUC value of logistic regression model.
