# Supplementary figures and images for: To infer the probability of cervical ossification of the posterior longitudinal ligament and explore its impact on cervical surgery
Source: Sci Rep. 2023 Jun 17;13:9816. doi: 10.1038/s41598-023-36992-7 (PMC10276809; doi:10.1038/s41598-023-36992-7)

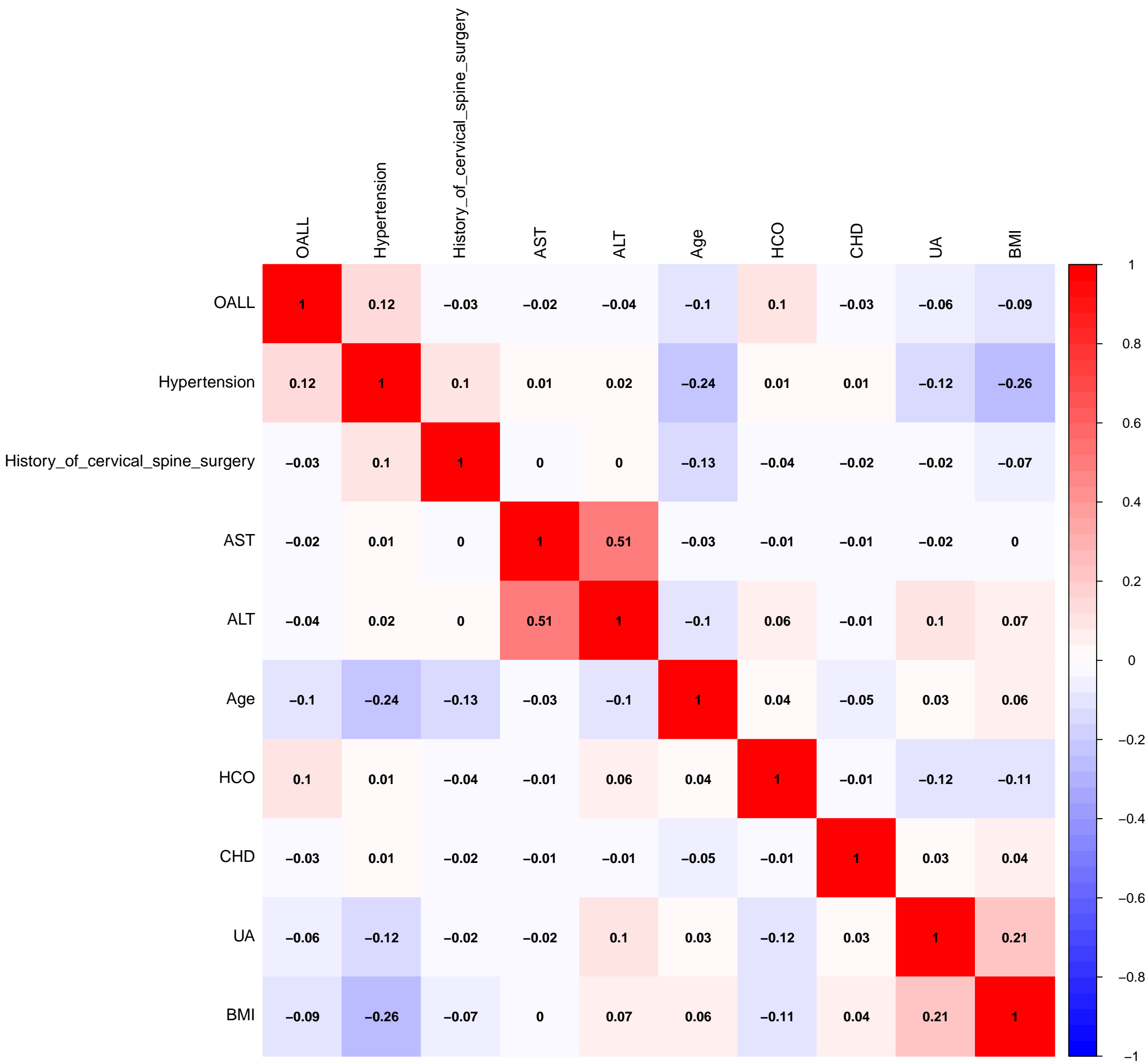

Supplement: Supplementary file 2 — Supplementary Information 2. [file 41598_2023_36992_MOESM2_ESM.pdf]

AUC= 0.6830692

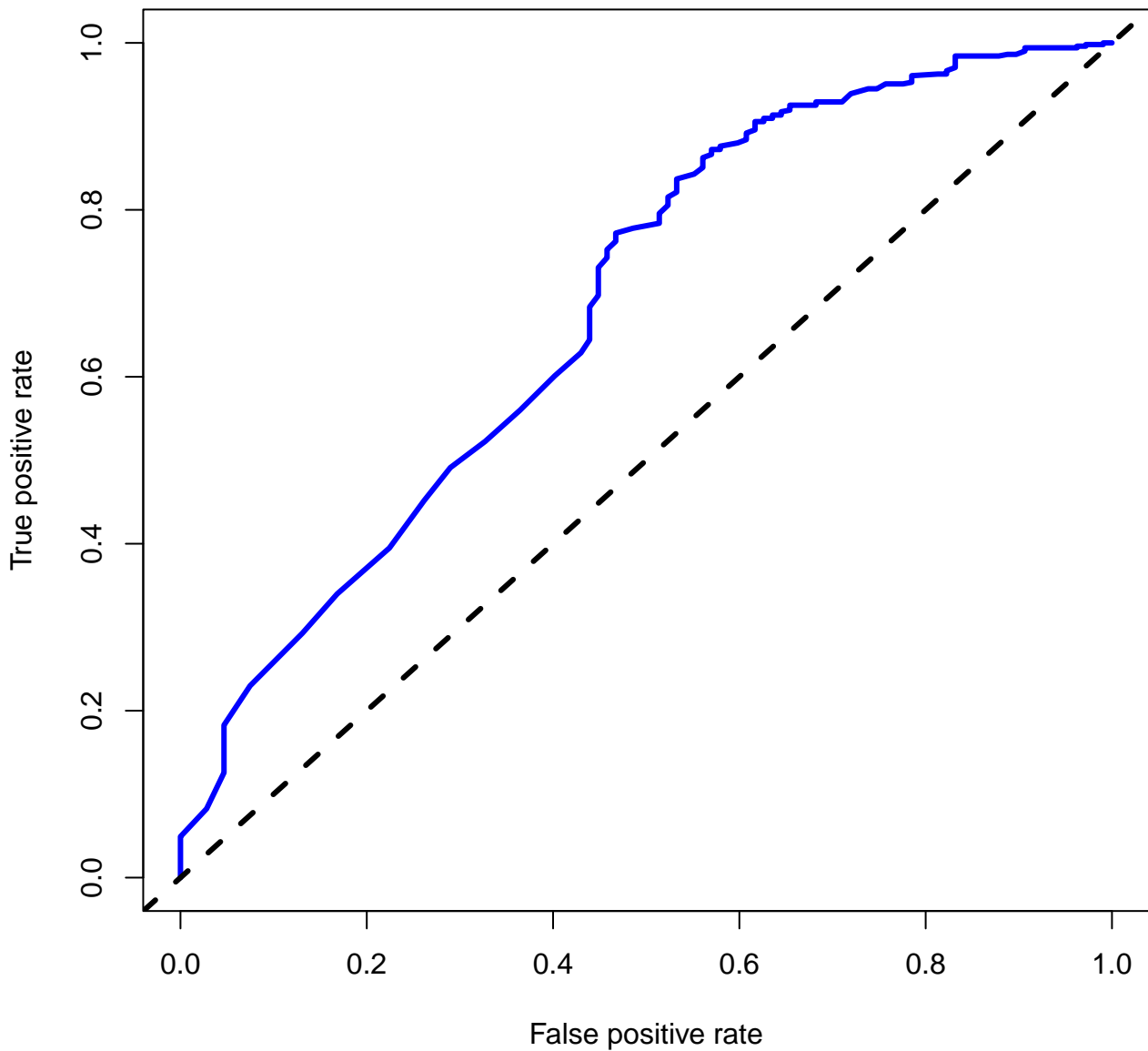

Supplement: Supplementary file 6 — Supplementary Information 6. [file 41598_2023_36992_MOESM6_ESM.pdf]
