# Supplementary material for: To infer the probability of cervical ossification of the posterior longitudinal ligament and explore its impact on cervical surgery
Source: Sci Rep. 2023 Jun 17;13:9816. doi: 10.1038/s41598-023-36992-7 (PMC10276809; doi:10.1038/s41598-023-36992-7)
Supplement: Supplementary file 4 — Supplementary Information 4. [file 41598_2023_36992_MOESM4_ESM.pdf]

广西医科大学第一附属医院
THE FIRST AFFILIATED HOSPITAL OF
GUANGXI MEDICAL UNIVERSITY

FIRST AFFILIATED HOSPITAL of GUANGXI MEDICAL UNIVERSITY
ETHICAL REVIEW COMMITTEE
Approval Notice

Approval Number: 2023-E018-01

Title: To infer the probability of cervical ossification of the posterior longitudinal ligament (OPLL) using machine learning method and explore its impact on cervical surgery——Based on clinical big data of cervical surgery.

Research Contents: The patients who underwent cervical spine surgery from 2012 to 2021 at the First Affiliated Hospital of Guangxi Medical University were selected. We collected 775 patients undergoing cervical spine surgery in the First Affiliated Hospital of Guangxi Medical University, and 84 variables were collected. There were 144 patients with cervical OPLL and 631 patients without cervical OPLL. They were randomly assigned to a training cohort and a validation cohort. A variety of machine learning (ML) methods were used to screen the variables and finally construct the diagnostic model. Finally, the cervical OPLL positive and negative postoperative patients were compared.

Applicant: liu chong

Application Department: Department of Spine and Osteopathy Ward

Date of Application: January 16, 2023

Date of Approval: January 19, 2023

Conclusion: We constructed a diagnostic model of cervical OPLL using the ML method and analyzed the postoperative differences in OPLL-positive patients. Our findings provide new techniques and insights for streamlining the diagnosis, treatment, and research direction of OPLL in the future.

Signature: ______________________

(Vice) Director of Ethical Review Committee

First Affiliated Hospital of Guangxi Medical University

Date: __January 19, 2023__

地 址 ： 广 西 南 宁 市 双 拥 路 6 号 邮 编 :530021 电话:0771-5350895
NO.6 SHUANGYONG ROAD NANNING GUANGXI P . C : 530021 TEL:+86-771-5350895
